# Destigmatizing Migraine

**DOI:** 10.7759/cureus.2711

**Published:** 2018-05-30

**Authors:** George Koshy Vilanilam, Mohammed K Badi, James F Meschia

**Affiliations:** 1 Neurology, Mayo Clinic Jacksonville, Fl, Jacksonville, USA

**Keywords:** migraine, chronic migraine, headache, stigma, social stigma, public health, destigmatization, patient advocacy, chronic neurological disorders with episodic manifestations, cdem

## Abstract

A migraine is one of the most disabling diseases in adults globally. There has been some progress in assessing various quantitative components of quality of life while we struggle to discuss the less addressed, albeit important, qualitative aspects, such as the stigma associated with this disease. People with a migraine can be viewed negatively by society. Victims of an invisible disease, they can feel dismissed by spouses, society, and physicians who may convey the sense that their disease is insignificant. There is an emergent need to promote the destigmatization and offer an enriched understanding at the level of both patients and healthcare providers.

## Editorial

A migraine is among the top-10 most-disabling conditions among adults worldwide. For years, the effect of migraines on quality of life (QoL) has been well documented. While different scales have been used to quantify its effects on QoL (Table 1) [1], little research has been done to assess the qualitative aspects of migraines; notably, the domain of the social stigma surrounding it and, by extension, an approach to rectify it.

**Table 1:** Scales quantifying Quality of Life (QoL) in persons with migraine

QoL Scales	Components Quantified
Stigma Scale for Chronic Illness (SSCI)	Impact and degree of the stigma
	Self/internalized stigma (e.g. feeling a sense of shame or anxiety)
	Enacted stigma (e.g. discrimination)
Short form of medical outcomes health survey (SF-12)	Physical health (e.g. pain limiting activities of daily living)
	Mental health (e.g. anxiety or depression limiting activities of daily living)
Migraine Disability Assessment Scale (MIDAS)	Days of headache-related disability over a three-month period
	Headache severity
Ability to work score	Capacity to work with their current condition

Stigma is a recognized social concept that describes a characteristic, trait, or diagnosis that is used to disrepute an individual. Stigma leads to a significant amount of prejudice, discrimination, and loss of status [1]. The stigmatizing effects of some diseases, like depression and epilepsy, are known to cause disrupted social relationships and decreased QoL. Beyond the 17th century, people with migraines started to be represented as privileged and self-absorbed individuals [2]. Interestingly, this view took another turn by the 19th century, when a migraine was perceived as a weakness of women of lower socio-economic status [2]. This gender difference in perception seems to line up with the fact that the prevalence of migraines is about three times in women than in men. Additionally, women have more frequent, longer lasting, and more severe headaches than men [3]. Therefore, it is hard not to wonder if epidemiological research has contributed in some way to the changing views of women in society.

Further, physicians caring for persons with a migraine were ridiculed as enabling and incompetent practitioners who encouraged their patients’ neurotic inclinations [2]. This paved the way for a considerably negative and gender-stereotyped view of the person with a migraine, which has persisted in varying intensities through decades. Unfortunately, stigma still follows migraines. An exploratory study using a focus group found that participants verbalized an unwillingness to tell others when they were experiencing a migraine, reporting that people were unsympathetic. “I think people look at you like, ‘Yeah, right, everybody has headaches. They are not that bad; just get a grip and keep going.’” Participants’ comments suggest that others’ reactions to migraines are mostly associated with anger and frustration. The consensus was that most people dismissed migraines as insignificant and assumed that patients with a migraine were exaggerating their symptoms [4]. The Chronic Migraine Epidemiology and Outcomes study threw light on the fact that more than 75% of spouses of patients with a chronic migraine did not believe them about their headaches [2].

Sadly, this emotionally laden issue extends to treating physicians. People with a migraine reported feeling “dismissed” by physicians who did not appear to take complaints of their pain seriously. Specifically, some participants reported that they had endured years of frequent migraines since being told to “live with it” by a physician [4]. Additionally, in selected groups, physicians viewed patients as drug seekers and see patients with a migraine as not having a severe disease [2]. However, the possibility exists that patient recollections of physician interactions may not truly reflect the nature of their communications and may have been taken out of context [4].

**Figure 1:**
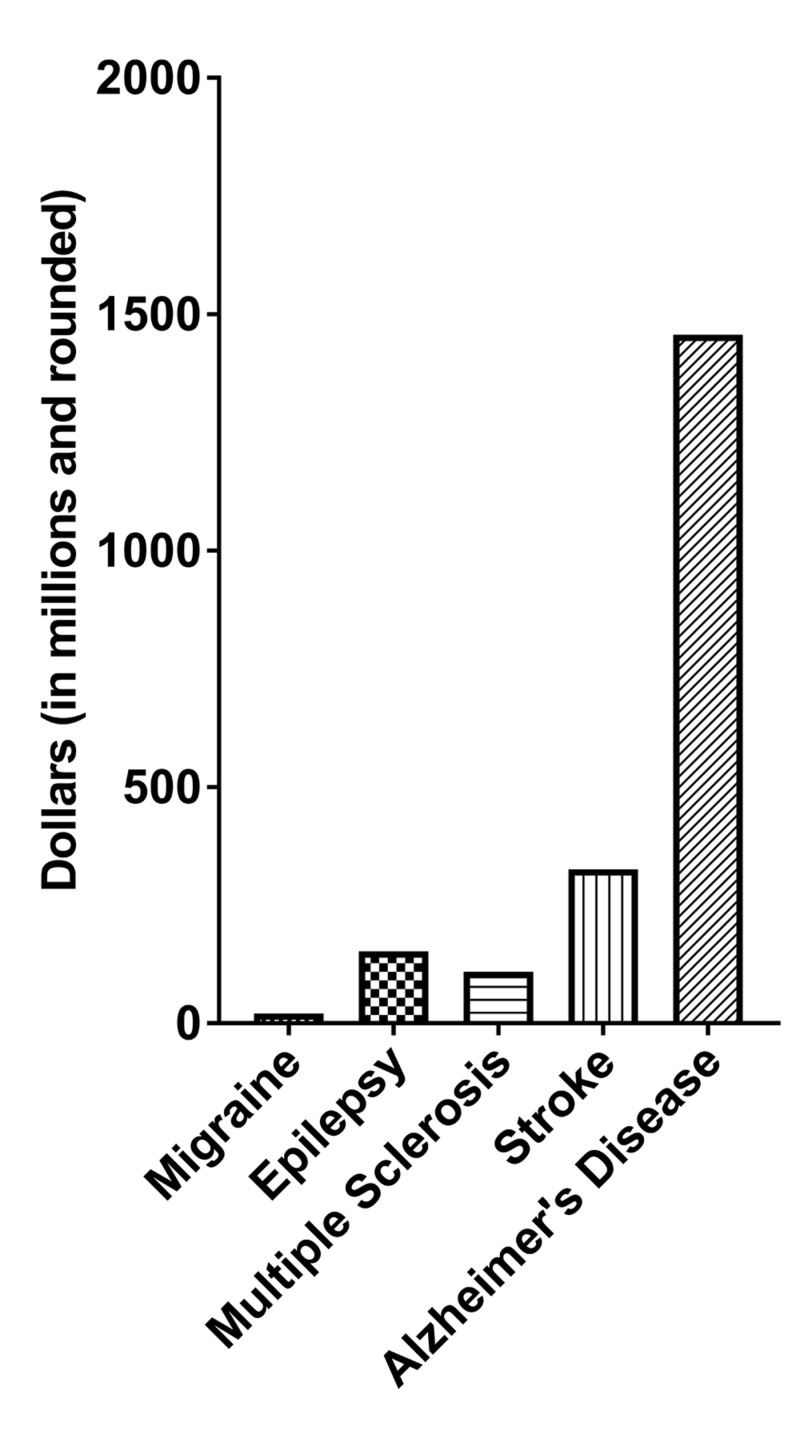
2018: estimated NIH categorical funding for migraine compared to other chronic neurological disorders The graph portrays the annual support level for selected neurological conditions based on grants, contracts, and other funding mechanisms used across the National Institutes of Health (NIH) data published by the National Center for Health Statistics (NCHS) at the Centers for Disease Control & Prevention (CDC).

Despite the high prevalence of migraines, there is a significant disparity toward research on this neurological disorder. In a study conducted to measure QoL among patients with migraines, it showed that persons with a chronic migraine experience more stigmas and disability, and are less likely to work when compared to persons with epilepsy [1]. It is, therefore, both intriguing and surprising to note that of the various chronic neurological disorders with episodic manifestations (CDEM), including migraines, epilepsy, and multiple sclerosis, the estimate of the National Institutes of Health (NIH) categorical funding for the year 2018 is still the lowest for migraines. Specifically, the projected NIH funding for migraines is about 21-million US dollars, which is a mere 1/7th of that for epilepsy (Figure 1) [5].

In light of past and recent evidence, there exists an emergent need to address the stigma surrounding migraine in these domains (family, doctors, and research), as this might facilitate an improved understanding of the perspectives of persons with migraines regarding their disease and treatment.

Moving forward

**Figure 2:**
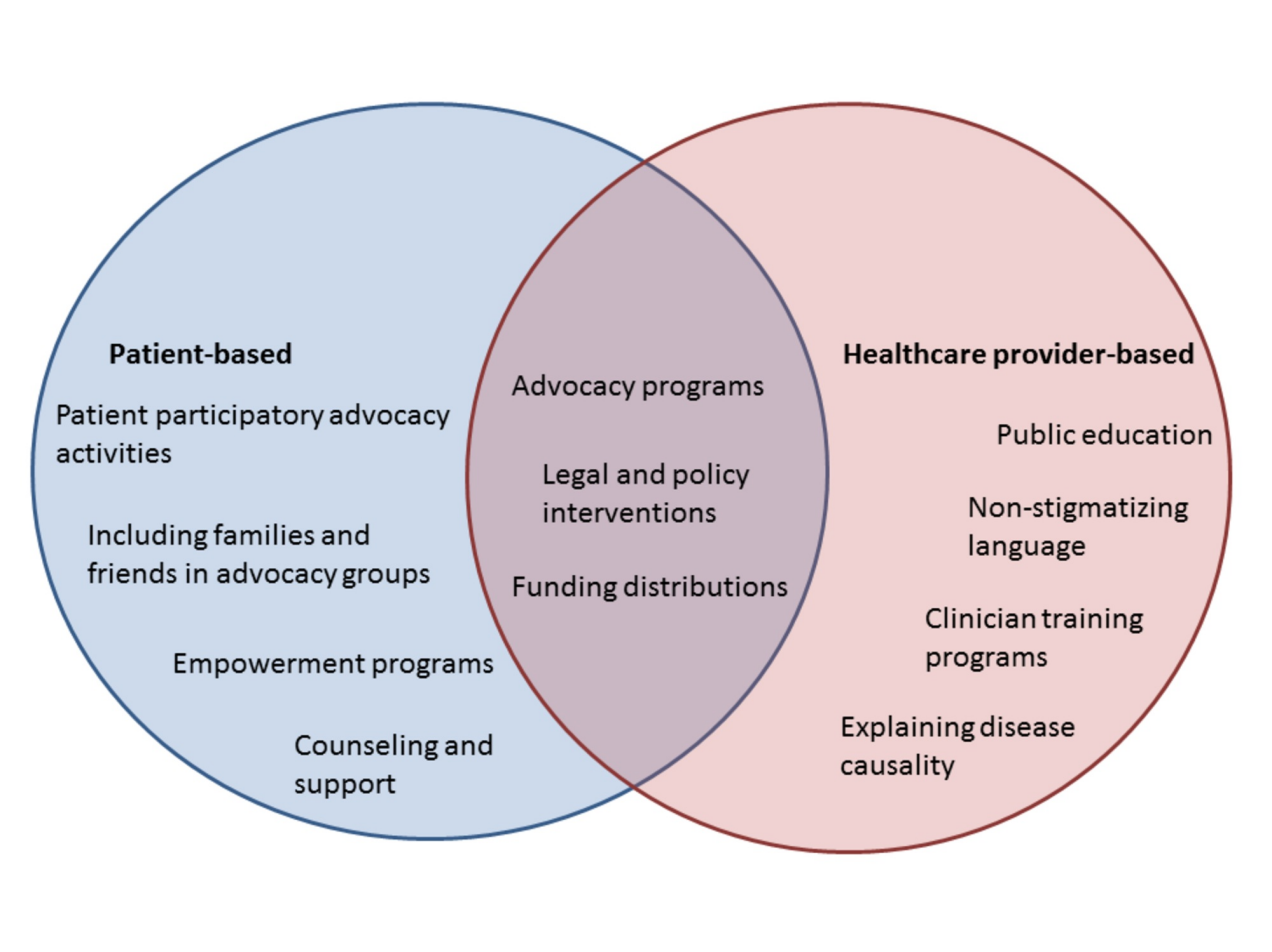
Roles proposed for patients and healthcare providers in destigmatizing migraine

Destigmatization needs to take place at the level of both patients and healthcare providers. At the level of patients, patient participatory advocacy activities (particularly ones where patients congregate such as walks, runs, and education camps) have proven to be instrumental in destigmatizing diseases. Moreover, the participation of people with migraines along with their families and friends is critical for migraine advocacy [2]. At the interpersonal level, counseling, therapy, and empowerment endeavors have been shown to benefit people with migraines [1]. At the level of healthcare providers, public education and employing causality-based (pathophysiology) descriptions of diseases make a difference. Patients want their physicians to demonstrate listening and taking their concerns seriously before offering treatment. Attempting to reflect the concerns that the patient has expressed and having asked the patient what he or she expects from treatment could initiate a mutually beneficial collaborative relationship: The patient feels understood and heard while the physician gains an accurate understanding of what the patient desires in treatment. Ultimately, the result may be a greater success with therapy [4]. Hence, there is also a need for clinicians to be trained on the use of non-stigmatizing language to describe the disease to their patients [2]. Additionally, patients and healthcare providers are instrumental in reshaping policies and funding distributions by working in conjunction on disease advocacy (Figure 2) [1].

As pharmacotherapy for migraines advances, we are in need of a concurrent movement to allocate our efforts to patient-centered outcomes. It is time we regard a migraine as the neurological disorder it is.
